# Evaluating the Perception of Undergraduate Medical Students About the Educational Environment by Using the Dundee Ready Educational Environment Measure (DREEM) Questionnaire

**DOI:** 10.7759/cureus.57245

**Published:** 2024-03-30

**Authors:** Gaurang N Algotar, Viral N Chauhan, Sanjay J Mehta

**Affiliations:** 1 Forensic Medicine, Gujarat Medical Education & Research Society Medical College, Navsari, IND; 2 Forensic Medicine, Gujarat Medical Education & Research Society Medical College, Morbi, IND; 3 Microbiology, Swaminarayan Institute of Medical Sciences & Research, Kalol, IND

**Keywords:** dreem survey, student perception, medical education, student satisfaction, educational environment

## Abstract

Background

This study uses the Dundee Ready Educational Environment Measure (DREEM) questionnaire to investigate undergraduate medical students' perceptions regarding their educational environment. The study recognizes the pivotal role of the educational environment in shaping future healthcare professionals and aims to contribute valuable insights for continuous improvement. The DREEM questionnaire, a validated tool, provides a structured approach to assess various dimensions of the educational environment. The study explores teaching and learning, academic atmosphere, student self-perception, social support, and overall satisfaction, seeking to identify strengths and areas for enhancement. The overarching goal is to offer evidence-based recommendations for academic institutions, curriculum developers, and policymakers to foster an environment that optimally nurtures the learning experiences of undergraduate medical students. The study aspires to contribute to the ongoing efforts to refine and elevate medical education, ensuring the holistic development of future healthcare professionals.

Aims & objectives

Using the DREEM questionnaire, evaluate undergraduate medical students' perceptions regarding their educational environment. Identifying the strengths and weaknesses in the current educational environment, encompassing teaching and learning, academic atmosphere, student self-perception, and social support.

Methodology

All undergraduate medical students of C.U.Shah Medical College and Hospital, Surendranagar, Gujarat (India), were included in the study. DREEM questionnaire was introduced in classroom settings in digital form with the help of Google Forms. The perceptions were obtained and analyzed with the help of Google Sheets.

Results

The DREEM questionnaire mean scores (124.58/200) indicate an overall positive perception of the educational environment among medical students, with total scores falling within the 'More Positive than Negative' range. The domain-wise analysis reveals scores for Students' Perception of Learning (SPL) 30.09/48, Students' Perception of Teachers (SPT) 27.87/44, Students' Academic Self-Perceptions (SASP) 20.60/32, Students' Perception of Atmosphere (SPA) 30.31/48, and Students' Social Self-Perceptions (SSSP) 15.72/28, indicating varying perceptions across different aspects of the educational environment. Within the domains, the SPL scores range from 10 to 44 (mean: 29.91), with one response in the 'very poor' range. SPT scores vary from 4 to 44 (mean: 27.49), with three 'very poor' responses. SASP scores range from 6 to 32 (mean: 20.73), with one response in the 'Feelings of total failure' range. SPA scores range from 9 to 48 (mean: 30.29), with one response in the 'Very poor environment' range. SSSP scores vary from 6 to 28 (mean: 15.66), with five 'Miserable' responses.

Conclusion

The study using DREEM scores highlights a generally positive perception of the educational environment among medical students. Areas for improvement include addressing fatigue-related concerns and enhancing teacher-student interactions, emphasizing the need for targeted interventions to ensure ongoing enhancement in the learning environment.

## Introduction

Medical education plays a pivotal role in shaping the future of healthcare professionals, and the educational environment in which students learn significantly influences their academic performance, well-being, and overall satisfaction with their educational experience [1-3]. The dynamic nature of medical education requires continuous assessment and improvement to ensure that it meets the evolving needs of students and the healthcare industry [1]. Recognizing the importance of understanding undergraduate medical students' perceptions regarding their educational environment, this research focuses on utilizing the Dundee Ready Educational Environment Measure (DREEM) questionnaire [4].

The DREEM questionnaire is a 50-item questionnaire developed by Roff et al. to measure the educational environment in health professional education programs [4]. The questionnaire was developed using a Delphi approach involving a range of health professional educators in different settings and countries [4]. As such, the DREEM questionnaire is reported to be appropriate for use within health professional programs, not just medicine, and is not culture or context-specific [4].

Each item is measured using a five-point Likert scale: 0 is strongly disagree, 1 is disagree, 2 is neither agree or disagree, 3 is agree, and 4 is strongly agree. Respondents are presented with a statement and asked to select a response. Items 4, 8, 9, 17, 25, 35, 39, 48, and 50 are negatively worded, and these require recoding before calculating the total and subscale scores.

The 50 items are divided into five subscales based on the initial psychometric analysis presented by Roff et al. The five subscales are Students' Perception of Learning (SPL), Students' Perception of Teachers (SPT), Students' Academic Self-perceptions (SASP), Students' Perception of Atmosphere (SPA), and Students' Social Self-perception (SSSP) [4]. The analysis of these subscales provides a detailed understanding of the strengths and challenges within the educational environment, allowing for targeted interventions and improvements in specific domains. In the discussion section, these interpretations are considered alongside the collected data to provide a nuanced analysis of the research findings.

This research seeks to explore the multifaceted dimensions of the educational environment, delving into aspects such as teaching and learning, academic atmosphere, student self-perception, social support, and overall student satisfaction [5-8]. Understanding how students perceive their learning environment is crucial for educational institutions to implement targeted interventions that enhance the quality of medical education and contribute to the holistic development of future healthcare professionals [6,9].

As this study is initiated, the overarching goal is to present evidence-based recommendations to academic institutions, curriculum developers, and policymakers to foster an environment that nurtures optimal learning experiences for undergraduate medical students [6,9]. By examining students' perceptions through the lens of the DREEM questionnaire, valuable insights are aspired to be contributed, supporting ongoing efforts to refine and elevate the educational journey for those who will shape the future of healthcare [4].

## Materials and methods

This study employed a cross-sectional survey design to comprehensively investigate the perceptions of all undergraduate medical students who were enrolled at C. U. Shah Medical College, Surendranagar-Gujarat (India), during the study period from March 2022 to July 2022. Before the initiation of the study, ethical approval was obtained from the Institutional Ethics Committee (IEC)- Human Research, C.U.Shah Medical College and Hospital, Surendranagar, under the letter number CUSMC/IEC(HR)/RP/6/2022/Final Approval/89/2022, dated 14/03/2022.

Utilizing a census approach, the study included the entire eligible student population. A total of 356 undergraduate students provided informed consent and were included in the study cohort. Students who did not provide consent or were absent during the administration of the DREEM questionnaire were excluded from the study sample.

Data were collected by administering the DREEM questionnaire, a validated instrument designed to assess various dimensions of the educational environment. The questionnaire, consisting of 50 items across five domains, was distributed electronically with the help of Google Forms, allowing participants to respond on a five-point Likert scale. A pilot test was conducted to enhance data quality, and the questionnaire's clarity and relevance were validated before the full-scale survey.

Descriptive statistics were employed to summarize the characteristics of the study cohort, including the academic year of the students enrolled.

Ethical considerations included obtaining informed consent from participants, ensuring anonymity and confidentiality in data handling, and disseminating only aggregated data to protect participant privacy.

## Results

A cohort comprising 356 undergraduate students volunteered and granted informed consent, thus becoming participants in the study. The distribution across academic years was as follows: 91 students from the I MBBS, 95 students from the II MBBS, 92 students from the III MBBS, and 78 students from the IV MBBS.

An item-wise score of the DREEM questionnaire is shown (Table 1). The total score for I MBBS is 125.97 (SD 17.66), II MBBS is 132.02 (SD 21.01), III MBBS is 130.40 (SD 18.80), IV MBBS 109.94 (SD 18.76) and the overall combined mean score is 124.58 (SD 21.81). Total scores are in the 'More Positive than Negative' range. On analysis of individual items, Item No. 4 (Domain SSSP; Question: I am too tired to enjoy the course) has a 1.84 Mean, item No. 8 (Domain: SPT; Question: The teachers ridicule the students) has a 1.91 Mean (SD 1.04), item No. 9 (Domain: SPT; Question: The teachers are authoritarian) has 1.67 Mean (SD 0.94), item No. 25 (Domain: SPL; Question: The teaching over-emphasizes factual learning) has 1.54 Mean (SD 0.82) and item No. 48 (Domain: SPL; Question: The teaching is too teacher-centered) has 1.74 Mean (SD 0.89); which have mean scores of 2 or less, which is considered as a problematic area.

**Table 1:** Academic year-wise DREEM questionnaire mean score (n=356) SPL: Students’ Perception of Learning; SPT: Students’ Perception of Teachers; SASP: Students’ Academic Self-Perceptions; SSSP: Students’ Social Self-Perceptions; SPA: Students’ Perception of Atmosphere

Domain	No.	Item	Item-wise Mean score					
			I MBBS (n=91)	II MBBS (n=95)	III MBBS (n=92)	IV MBBS (n=78)	Mean	SD
SPL	1	I am encouraged to participate in class	3.01	2.84	2.99	2.56	2.86	0.76
SPT	2	The teachers are knowledgeable	3.20	3.29	3.33	2.69	3.15	0.77
SSSP	3	There is a good support system for students who get stressed	2.30	2.48	2.40	1.58	2.22	1.07
SSSP	4	I am too tired to enjoy the course	1.69	2.01	1.92	2.33	1.84	1.01
SASP	5	Learning strategies that worked for me before continue to work for me now	2.35	2.54	2.42	2.09	2.36	0.84
SPT	6	The teachers deliver research-led teaching	2.78	2.81	2.83	1.78	2.58	0.88
SPL	7	The teaching is often stimulating	2.68	2.68	2.83	2.09	2.59	0.78
SPT	8	The teachers ridicule the students	1.85	2.08	1.89	2.42	1.91	1.04
SPT	9	The teachers are authoritarian	1.62	1.89	1.60	2.28	1.67	0.94
SASP	10	I am confident about passing this year	3.31	3.45	3.00	2.90	3.18	0.90
SPA	11	The atmosphere is relaxed during laboratory/practical/fieldwork classes	2.33	2.86	2.63	2.08	2.49	1.01
SPA	12	The course is well timetabled	2.58	2.88	3.16	1.76	2.63	1.05
SPL	13	The teaching is student-centred	2.70	2.79	2.64	1.99	2.55	0.88
SSSP	14	I am rarely bored on this course	2.13	2.15	2.14	1.77	2.06	1.01
SSSP	15	I have good friends in this faculty	2.67	2.57	2.49	2.36	2.53	0.93
SPL	16	The teaching helps to develop my competence	2.97	2.83	2.99	2.17	2.76	0.82
SPA	17	Cheating is a problem in this faculty	2.58	2.80	2.76	2.51	2.60	0.97
SPT	18	The teachers help me to develop my practical skills	2.88	2.83	3.16	2.27	2.81	0.82
SSSP	19	My social life is good	2.64	2.76	2.63	2.01	2.53	1.06
SPL	20	The teaching is well-focused	2.93	2.78	2.79	2.05	2.66	0.89
SASP	21	I feel I am being well-prepared for my career	2.92	3.09	2.70	2.09	2.73	0.95
SPL	22	The teaching helps to develop my confidence	2.75	2.94	2.95	2.13	2.71	0.86
SPA	23	The atmosphere is relaxed during lectures	2.33	2.82	2.63	2.03	2.47	0.95
SPL	24	The teaching time is put to good use	2.55	2.69	2.64	1.77	2.44	0.95
SPL	25	The teaching over-emphasizes factual learning	1.43	1.42	1.49	2.44	1.54	0.82
SASP	26	Last year’s work has been a good preparation for this year’s work	2.62	2.74	2.65	2.35	2.60	0.84
SASP	27	I am able to memorize all I need	2.16	2.34	2.32	2.00	2.21	0.95
SSSP	28	I seldom feel lonely	2.15	2.11	2.05	1.97	2.08	1.06
SPT	29	The teachers are good at providing feedback to students	2.67	2.86	2.71	2.22	2.63	0.87
SPA	30	There are opportunities for me to develop my interpersonal skills	2.74	2.97	3.10	2.21	2.78	0.89
SASP	31	I have learned a lot about the way scientific research is carried out	2.38	2.40	2.35	1.72	2.23	1.03
SPT	32	The teachers provide constructive criticism here	2.74	2.80	2.72	2.12	2.61	0.86
SPA	33	I feel comfortable in class socially	2.82	2.88	2.83	2.55	2.78	0.78
SPA	34	The atmosphere is relaxed during seminars/tutorials	2.25	2.76	2.62	2.31	2.49	0.98
SPA	35	I find the experience disappointing	2.44	2.41	2.39	2.41	2.29	0.93
SPA	36	I am able to concentrate well	2.41	2.57	2.45	2.13	2.40	0.94
SPT	37	The teachers give clear examples	2.91	2.96	2.97	2.41	2.83	0.79
SPL	38	I am clear about the learning objectives of the course	2.78	2.81	2.88	2.38	2.73	0.85
SPT	39	The teachers get angry in class	1.99	2.05	2.12	2.37	2.02	0.98
SPT	40	The teachers are well-prepared for their classes	2.93	3.05	2.98	2.23	2.82	0.91
SASP	41	My problem-solving skills are being well developed here	2.71	2.72	2.70	2.04	2.56	0.91
SPA	42	The enjoyment outweighs the stress of the course	2.22	2.58	2.22	2.13	2.29	0.94
SPA	43	The atmosphere motivates me as a learner	2.63	2.69	2.91	2.06	2.60	0.94
SPL	44	The teaching encourages me to be an active learner	2.71	2.80	2.85	2.18	2.65	0.88
SASP	45	Much of what I have to learn seems relevant to a career in medical sciences	2.92	2.99	3.08	2.36	2.86	0.73
SSSP	46	My accommodation is pleasant	2.45	2.64	2.49	1.99	2.41	0.96
SPL	47	Long-term learning is emphasized over short-term learning	2.71	2.71	2.76	2.44	2.66	0.75
SPL	48	The teaching is too teacher-centred	1.74	1.91	1.82	2.37	1.74	0.89
SPA	49	I feel able to ask the questions I want	2.42	2.64	2.59	2.15	2.46	0.92
SPT	50	The students irritate the teachers	2.27	2.33	2.87	2.71	2.46	1.08

The academic year-wise total scores were derived from the DREEM questionnaire (Table 2). Within the various domains, the total score for SPL is 30.09 out of 48, SPT is 27.87 out of 44, SASP is 20.60 out of 32, SPA is 30.31 out of 48, and SSSP is 15.72 out of 28. Academic year-wise total scores expressed out of 200, vary across I MBBS (125.97), II MBBS (132.02), III MBBS (130.40), and IV MBBS (109.94). The mean total score for all academic years combined is 124.58 out of 200, reflecting an overall positive perception of the educational environment.

**Table 2:** DREEM score according to academic year (n=356) SPL: Students’ Perception of Learning; SPT: Students’ Perception of Teachers; SASP: Students’ Academic Self-Perceptions; SSSP: Students’ Social Self-Perceptions; SPA: Students’ Perception of Atmosphere; DREEM: Dundee Ready Educational Environment Measure

Domain	Max. Score	Domain-wise total score					
		I MBBS (n=91)	II MBBS (n=95)	III MBBS (n=92)	IV MBBS (n=78)	Mean Score	SD
SPL	48	30.97	31.2	31.62	26.56	30.09	5.37
SPT	44	27.84	28.97	29.16	25.5	27.87	5.27
SASP	32	21.38	22.26	21.21	17.54	20.60	4.36
SPA	48	29.75	32.87	32.28	26.32	30.31	6.65
SSSP	28	16.03	16.72	16.13	14.01	15.72	3.59
Total Score	200	125.97	132.02	130.40	109.94	124.58	21.81

A comprehensive breakdown of the domain-wise DREEM scores (Table 3). Within the SPL domain, the range of scores spans from 10 to 44, with a mean value of 29.91 and a standard deviation of 5.37. Notably, one response falls within the 'very poor' range (0-12). In the SPT domain, scores vary from 4 to 44, with a mean value of 27.49 and a standard deviation of 5.27. Three responses fall within the 'very poor' range (0-11). For the SASP domain, scores range from 6 to 32, with a mean value of 20.73 and a standard deviation of 4.36. One response falls into the 'Feelings of total failure' range (0-8). In the SAP domain, scores range from 9 to 48, with a mean value of 30.29 and a standard deviation of 6.65. One response falls within the 'Very poor environment' range (0-12). Finally, in the SSSP domain, scores vary from 6 to 28, with a mean value of 15.66 and a standard deviation of 3.59. Five responses fall within the 'Miserable' range (0-7).

**Table 3:** DREEM score according to academic year (n=356) DREEM: Dundee Ready Educational Environment Measure

Domain	Max. Score	Domain-wise total score				
		I MBBS	II MBBS	III MBBS	IV MBBS	Mean Score
Students’ Perception of Learning (SPL)	48	30.97	31.2	31.62	26.56	30.09
Students’ Perception of Teaching (SPT)	44	27.84	28.97	29.16	25.5	27.87
Students’ Academic Self-Perception (SASP)	32	21.38	22.26	21.21	17.54	20.60
Students’ Perception of Atmosphere (SPA)	48	29.75	32.87	32.28	26.32	30.31
Students’ Social Self-Perception (SSSP)	28	16.03	16.72	16.13	14.01	15.72
Total Score	200	125.97	132.02	130.40	109.94	124.58

## Discussion

The results obtained from administering the DREEM questionnaire provide valuable insights into the perceived educational environment across different academic years in the medical curriculum. The discussion will be structured to address specific aspects revealed by the DREEM scores and will draw upon relevant literature to provide context and support for the findings.

Overall educational environment

The aggregated DREEM score, representing the collective assessment of the educational environment, yielded a mean value of 124.58 out of a total possible score of 200, which indicates a more positive than negative perception of the educational environment among medical students [10, 11]. This aligns with the general trend observed in other medical schools globally.

Domain-wise analysis

SPL Domain

The SPL domain score of 30.09 (SD 5.37) out of 48 suggests a generally positive view of the learning experience. However, the mean score of 1.54 for item No. 25 ("The teaching over-emphasizes factual learning") indicates a potential area of concern [12].

SPT Domain

In the SPT domain, the score of 27.87 (SD 5.27) out of 44 indicates a favorable perception. However, items such as No. 8 ("The teachers ridicule the students") and No. 9 ("The teachers are authoritarian"), with mean scores of 1.91 and 1.67, respectively, draw attention to aspects of the teacher-student relationship that may require further exploration and improvement [13,14].

SASP Domain

Within the SASP domain, the score of 20.60 (SD 4.36) out of 32 indicates a moderately positive perception. However, a response falling into the 'Feelings of total failure' range for one item emphasizes the importance of addressing students' self-perception concerns, which can impact motivation and academic achievement [15].

SPA Domain

In the SPA domain, the score of 30.31 (SD 6.65) out of 48 reflects a generally positive view. However, one response falling within the 'Very poor environment' range highlights the need for targeted interventions to enhance the overall educational atmosphere [7].

SSSP Domain

The Student's Social Self-Perception (SSSP) domain, with a score of 15.72 (SD 3.59) out of 28, suggests a relatively positive social environment. Nevertheless, the presence of five responses falling within the 'Miserable' range for individual item point to specific social aspects that may need attention [16].

Academic year-wise analysis

The variation in total DREEM scores across different academic years (I MBBS, II MBBS, III MBBS, and IV MBBS) provides valuable insights into the evolving perceptions of students. The higher scores in II MBBS and III MBBS, compared to I MBBS, may indicate students' adaptation to the educational environment over time [17]. Comparisons with similar studies, such as those conducted in India [18,19] and other countries [20,21], reinforce the notion that certain challenges and strengths in medical education environments are universal.

Strengths and weaknesses of the current education environment

Strengths

Overall Positive Perception: The total combined DREEM score indicates an overall positive perception of the educational environment among medical students, with scores falling in the 'More Positive than Negative' range. Domain-wise Strengths: Specific domains such as SPL and SPA (Student's Perception of Atmosphere) reflect positive views, suggesting effective learning experiences and a favorable overall atmosphere. Academic Year Variation: The variation in total scores across academic years suggests an adaptability and potential improvement over time, with higher scores in II MBBS and III MBBS. Global Comparison: Comparisons with similar studies globally support the idea that certain challenges and strengths in medical education environments are universal.

Weaknesses

Specific Item Concerns: Items such as "I am too tired to enjoy the course" and "The teachers ridicule the students" highlight specific concerns within the SSSP and SPT domains, indicating areas that need attention. Domain-wise Challenges: Within the SASP (Student's Academic Self-Perception) and SSSP domains, responses fall into the 'Feelings of total failure' and 'Miserable' ranges, respectively, suggesting challenges in students' self-perception and social aspects. Teacher-Student Relationship: Items related to teachers being perceived as authoritarian and ridiculing students indicate potential issues in the teacher-student relationship.

Solutions

Addressing Specific Item Concerns: Targeted interventions should address specific concerns raised by items with mean scores of 2 or less, such as fatigue and negative teacher-student interactions. Enhancing Self-Perception Support: Implement interventions to enhance students' self-perception, focusing on areas like academic self-perception and feelings of failure, to positively impact motivation and academic achievement. Improving Teacher-Student Relationship: Initiatives to improve the teacher-student relationship, including communication and feedback mechanisms, can address concerns related to teachers being perceived as authoritarian or ridiculing students. Qualitative Exploration: Incorporating qualitative research methods can provide deeper insights into the reasons behind certain perceptions, enabling a more nuanced understanding of the educational environment. Continuous Monitoring and Feedback: Establish a system for continuous monitoring and feedback to address evolving challenges and build on strengths over time.

Limitations of the study

The study's limitations are underscored by its lack of an in-depth exploration of the problematic areas. Relying solely on quantitative parameters and a cross-sectional design may not capture the nuanced aspects essential to comprehensively understanding students' perceptions of the educational environment.

## Conclusions

The comprehensive analysis of the DREEM questionnaire across various academic years in our medical curriculum offers valuable insights into the perceived educational environment. The overall positive perception, as indicated by a combined DREEM score of 124.58 out of 200, aligns with global trends in medical schools. Strengths observed in specific domains, particularly in learning experiences (SPL) and the overall educational atmosphere (SPA), suggest commendable aspects of our educational environment.

However, the study highlights specific challenges that demand attention. Concerns regarding students' tiredness affecting their course enjoyment (SSSP) and negative perceptions of teacher-student relationships (SPT) indicate areas requiring immediate intervention. Furthermore, variations in scores across academic years emphasize the importance of ongoing evaluation and enhancement.

Targeted interventions are recommended to address these challenges. Initiatives to reduce fatigue, improve teacher-student interactions, and enhance students' self-perception are essential. Qualitative research methods can complement these efforts, providing a deeper understanding of the underlying issues and facilitating a more contextually informed approach.

The study underscores the need for a holistic strategy, combining quantitative assessments with qualitative exploration and continuous monitoring. By implementing these recommendations, we can further elevate the medical education environment, ensuring it nurtures positive learning experiences and supports the holistic development of our students.
